# Casirivimab-imdevimab neutralizing SARS-CoV-2: post-infusion clinical events and their risk factors

**DOI:** 10.1186/s40780-021-00233-8

**Published:** 2022-01-03

**Authors:** Junichi Yoshida, Kenichiro Shiraishi, Tetsuro Tamura, Kazuhiro Otani, Tetsuya Kikuchi, Akiko Mataga, Takako Ueno, Masao Tanaka

**Affiliations:** 1 Infection Control Committee, Shimonoseki City Hospital, 1-13-1 Koyo-cho, Shimonoseki, 750-8520 Japan; 2 Department of Medicine and Biosystemic Science, Kyushu University Faculty of Medicine, 3-1-1 Maidashi, Higashi-ku, Fukuoka, 812-8582 Japan

**Keywords:** SARS-CoV-2, Casirivimab, Imdevimab, Fever, Hypoxia

## Abstract

**Background:**

Casirivimab-imdevimab has been developed to neutralize SARS-CoV-2. The global clinical trials in outpatients documented several adverse effects (AE), which mandate caution in Japan where part of patients return home. To investigate post-infusion clinical events and their risk factors, we attempted a retrospective study.

**Main body:**

Subjects were a consecutive series of inpatients with COVID-19 undergoing an infusion of casirivimab-imdevimab in our institute. The criteria for administration were in accordance with previous clinical trials, e.g., exclusion of patients necessitating oxygen supply. In Japan, however, SARS-CoV-2 vaccinees were eligible.

Methods were review of background factors of status, imaging, and laboratory findings for the outcome of post-infusion events such as temperature increase (Temp+), pulse oximetry below 94%, and other events. Also, we documented the drug efficacy.

Of a total of 96 patients with a median follow-up of 54 days, one (1.0%) died who alone was an exception demanding oxygen supply. Other 95 patients (99.0%) recovered from fever and hypoxia by Day 4 and later had no worsening of COVID-19.

Median increase of body temperature was 1.0 degrees Celsius, which was used for computation of Temp+. Multivariate analysis showed that for Temp+ (*n* = 47), white blood cell counts more than 4.3 × 10^3^/microliter (Odds Ratio [OR] 2.593, 95% Confidence Interval [CI] 1.060–6.338, *P* = 0.037) was at risk, whereas 2-time vaccination for SARS-CoV-2 (OR 0.128, 95% CI 0.026–0.636, *P* = 0.012) was a preventing factor. Likewise for lowered oximetry (*n* = 21), CT showing bilateral ground glass attenuation (OR 5.544, CI 1.599–19.228, *P* = 0.007) was a significant risk factor. Two patients (2.1%) showed bradycardia (asymptomatic, intervention not indicated) on Day 3 and recovery on Day 5.

Limitations for this study included the difficulty distinguishing AE from worsening of COVID-19, thus we documented as clinical events.

**Conclusions:**

For 24 h after infusion of casirivimab-imdevimab, COVID-19 patients with increased white blood cell counts may be predisposed to temperature elevation more than 1.0 degrees centigrade, as may bilateral ground glass opacity to lowered oximetry. Thus, patients with leukocytosis and bilateral ground glass attenuation may need precaution for transient fever and hypoxia, respectively.

## Background

Casirivimab-imdevimab has been developed to neutralize the spike proteins of SARS-CoV-2 [1]. The clinical trials in COVID-19 outpatients showed its adverse effects (AE) including fever and hypoxia by day 4 as infusion-related reaction [1 at Supplementary Appendix]. Also, an independent data monitoring committee recommended halting another study in hospitalized patients supplemented with oxygen due to unfavorable risk/benefit [2]. In July 2021, however, the Japanese regulatory authority approved its use primarily in-hospital setting to monitor AE [3]. Post-infusion events, however, may reflect temporary worsening of COVID-19 indistinguishable from AE as patients recovering soon. Despite this difficulty, triage of patients eligible for early outpatient follow-up is urgently indicated. We investigated post-infusion clinical events and their risk factors retrospectively.

## Main body

Subjects were a consecutive series of inpatients positive for antigen or polymerase chain reaction of SARS-CoV-2 undergoing intravenous administration of casirivimab 600 mg and imdevimab 600 mg (Ronapreve 1332; Chugai Pharmaceuticals, Tokyo, Japan) in a single institute. The entry criteria were in accordance with previous clinical trials [1], where SARS-CoV-2 vaccinees were excluded. The Japanese regulatory agency [3], however, did not exclude previous vaccination in the domestic criteria. The Japanese agency described an entry criterion of body mass index more than 30 kg/m^2^, but we observed that of more than 25 kg/m^2^ by US Food and Drug Administration [4].

Methods were retrospective review of background factors for the outcome of 24-h post-infusion temperature course. Patients received supplemental oxygen whenever pulse oximetry fell below 94% but were removed when recovered.

The background factors included (i) demographics such as age, gender and body mass index (BMI), (ii) days from the symptom onset to the administration, (iii) body temperatures, (iv) pulse oximetry before and 24-h after infusion, (v) pre-infusion hematological and biochemical results, (vi) computed tomography (CT) showing ground glass attenuation in the bilateral lung, and (vii) the number of SARS-CoV-2 vaccination.

The primary endpoint of body temperature increase (Temp+) within 24-h post-infusion was defined as temperature increase more than the median difference from peak temperature to pre-infusion temperature. We specified the duration as 24-h based on frequent definition of “infusion reaction” within 24 h, whereas the clinical trial defined “infusion-related reaction” until the fourth day [1].

Secondary endpoints included pulse oximetry decrease below 94 within 24 h and any other AE, such as anaphylactoid reaction, arrhythmia, and biochemical abnormality. Also, we observed worsening COVID-19, such as prolonged supplementary oxygen more than 24-h, invasive ventilation, and death.

Statistical analyses included receiver-operating characteristics (ROC) analysis to determine cutoff values, using the ROC curve on the plot of sensitivity versus (1-specificity). Variables with *P* values of 0.05 or less at univariate logistic regression analysis underwent multivariate analysis. Statistical significance was defined as P less than 0.05. For computation, we used SPSS Version 26 (IBM Japan, Tokyo, Japan).

As for results, casirivimab-imdevimab was given from 2 August 2021 through 16 October 2021, when the delta variant of SARS-CoV-2 prevailed across the country [5]. Thus, all the subjects fulfilled the follow-up duration of 29 days or more such as in the clinical trial [1]. Of a total of 96 inpatients subjected, the median age was 55.5 while men accounted for 51 and women for 45 (Table 1). The ROC analyses showed that the age factor had a cutoff of 55, as did BMI that of 23.25 kg/m^2^ (Table 1). Body temperature increased by a median of 1.0 degrees Celsius, with which we calculated for Temp+. Only one patient receiving oxygen supply (1.0%) failed to meet the entry criteria [1] for compassionate use.

**Table 1:** Profile of background factors by temperature increase and oximetry decrease 24-h post-infusion of casirivimab-imdevimab

		Total		Temp+		Ox94	
Factor	Division (Unit)	*N* = 96	Median (Interquartile range)	*N* = 47	Median (Interquartile range)	*N* = 21	Median (Interquartile range)
Sex	Male	51		29		13	
	Female	45		18		8	
Smoking	Yes	59		29		12	
	Never	37		18		9	
CT Bilateral	Bilateral	34		21		15	
	Otherwise	62		26		6	
2-Time Vaccination	Yes	15		2		2	
	No	81		45		19	
Age	(Years)		55.5 (46.5–63.0)		53.0 (45.0–61.0)		56.0 (51.0–67.0)
Body MassIndex	(kg/m^2^)		23.25 (20.75–25.55)		24.30 (20.30–26.90)		24.80 (22.65–26.75)
Symptomatic	(Day)		3.0		3.0 (2.0–4.0)		4.0 (1.0–5.0)
PreTemp	Centigrade		36.9 (36.50–37.30)		36.7 (36.50–37.30)		36.70 (36.50–37.25)
PreOx	(%)		97.0		97.0 (96.0–97.0)		96.0 (96.0–97.0)
WhileBlood Cell	(×10^3^)		4.62		4.96 (3.99–5.92)		4.79 (3.88–6.24)
Neutrophil	(%)		65.70		66.60 (57.60–71.4)		67.90 (54.60–74.10)
Lymphocytes	(%)		23.95		22.90 (18.30–31.90)		23.60 (20.00–36.25)
C-reactive protein	(mg/dl)		0.69		0.61 (0.21–2.91)		3.06 (0.47–5.77)
Total Bilirubin	(mg/dl)		0.60		0.60 (0.40–0.70)		0.60 (0.50–0.75)
Creatinine	(mg/dl)		0.810		0.860 (0.74–1.00)		0.810 (0.72–1.02)
Platelet	(×10^3^)		186.5		185.0 (154.0–231.0)		184.0 (140.0–223.5)

Temp+, temperature increase more than 1.0 degrees Celsius; Ox94, post-infusion pulse oximetry below 94%; CT Bilateral, computed tomography showing bilateral ground glass attenuation; PreTemp, pre-infusion body temperature; PreOx, pre-infusion pulse oximetry

Multivariate analysis showed that for the primary endpoint Temp+ (*n* = 46, 47.9%), white blood cell counts more than 4.3 × 10^3^/microliter (Odds Ratio [OR] 2.593, 95% Confidence Interval [CI] 1.060–6.338, *P* = 0.037) was at risk whereas 2-time vaccination for SARS-CoV-2 (OR 0.128, 95% CI 0.026–0.636, *P* = 0.012) was a preventing factor (Table 2).

**Table 2:** Logistic regression analysis for factors influencing temperature increase more than 1.0 C 24-h post-infusion of casirivimab-imdevimab

		Univariate Analysis				Multivariate Analysis			
Factor	Cutoff	OR	CI, lower	CI, upper	P	OR	CI, lower	CI, upper	P
Age	55	1.8	0.802	4.042	0.154				
Male		1.977	0.876	4.463	0.101				
Smoking		1.020	0.448	2.322	0.962				
Body Mass Index	23	2.166	0.955	4.913	0.064				
Symptomatic	3 days	0.976	0.425	2.241	0.954				
CT Bilateral		2.237	0.95	5.265	0.065				
PreTemp	37	0.695	0.307	1.578	0.385				
PreOx	96%	1.646	0.434	6.250	0.464				
White Blood Cell	4.3 × 10^3^	2.412	1.050	5.537	0.038*	2.593	1.060	6.338	0.037*
Neutrophil	64%	1.415	0.631	3.174	0.400				
Lymphocyte	24%	0.778	0.349	1.736	0.540				
C-Reactive Protein	0.53 mg/dl	0.852	0.381	1.907	0.697				
Total Bilirubin	0.55 mg/dl	4.390	0.881	21.881	0.071				
Creatinine	0.7 mg/dl	2.912	1.157	7.331	0.023*	2.217	0.822	5.981	0.116
Platelet	180 × 10^3^	1.095	0.490	2.446	0.824				
2-Time Vaccination		0.123	0.026	0.581	0.008*	0.125	0.025	0.623	0.011*

CI, 95% confidence interval; CT Bilateral, computed tomography showing bilateral ground glass attenuation; PreTemp, pre-infusion body temperature; PreOx, pre-infusion pulse oximetry; *, *P* < 0.05

As to the secondary endpoints for lowered oximetry (*n* = 21), bilateral ground glass attenuation (OR 5.544, CI 1.599–19.228, *P* = 0.007) was a significant risk factor (Table 3).

**Table 3:** Logistic regression analysis on background factors influencing pulse oximetry below 94% 24-h after infusion of casirivimab-imdevimab

		Univariate Analysis				Multivariate Analysis			
Factor	Cutoff	OR	CI, lower	CI, upper	P	OR	CI, lower	CI, upper	P
Age	55	0.692	0.261	1.837	0.46				
Male		1.582	0.588	4.259	0.364				
Smoking		0.794	0.297	2.122	0.646				
Body Mass Index	23	3.467	1.152	10.431	0.027*	3.145	0.958	10.326	0.059
Symptomatic	3 days	2.338	0.874	6.255	0.091				
CT Bilateral		7.368	2.501	21.705	< 0.001*	5.544	1.599	19.228	0.007*
PreTemp	37	0.672	0.243	1.856	0.443				
PreOx	96%	2.706	0.686	10.669	0.155				
White Blood Cell	4300	0.992	0.373	2.638	0.988				
Neutrophil	64%	1.106	0.416	2.936	0.840				
Lymphocyte	24%	0.692	0.261	1.837	0.460				
C-Reactive Protein	0.53 mg/dl	3.286	1.092	9.888	0.034*	1.543	0.402	5.923	0.528
Total Bilirubin	0.55 mg/dl	1.134	0.222	5.796	0.880				
Creatinine	0.7 mg/dl	2.125	0.646	6.987	0.215				
Platelet	180 × 10^3^	0.912	0.346	2.405	0.853				
2-Time Vaccination		0.502	0.104	2.425	0.391				

CI, 95% confidence interval; CT Bilateral, computed tomography showing bilateral ground glass attenuation; PreTemp, pre-infusion body temperature; PreOx, pre-infusion pulse oximetry; *, *P* < 0.05

These events, fever and lowered oximetry, recovered by Day 5 in 95 patients (99.0%) except in the patient with compassionate use, who died on Day 11 with pre-existing severe heart failure. Bradycardia was noted in two patients (2.1%), 24-year-old male with smoking risk and 41-year-old male at risk of diabetes mellitus. Both of them demonstrated Grade 1 (asymptomatic, intervention not indicated [6]) on Day 3 and recovery on Day 5. Grade 3 hyponatremia [6] was developed in one patient (1.0%) on Day 6 but recovered on Day 9, who had pre-existing hyponatremia (Grade 2) by hydrochlorothiazide. Anaphylactoid reactions were not documented.

Apart from COVID-19, two patients had preexisting co-morbidity in need of antimicrobials. One of them had acute appendicitis which subsided on Day 3; the other had aspiration pneumonia with defervescence on Day 2.

Our study revealed that 24-h after infusion of casirivimab-imdevimab, (1) Temp+ was related to elevated white blood cell counts and (2) hypoxia was to bilateral ground glass attenuation. Temp+ in (1) may have relation with cytokine production activated by antibody agents such as casirivimab-imdevimab. As Liu et al. [7] described, increased white blood cell counts may represent later course of COVID-19, but our multivariate analysis failed to show relation with symptomatic days. In our series, Temp+ by 1.0 degrees centigrade had a high rate of 47.9% while a direct comparison with the clinical trial in outpatient setting [1] is difficult. Our patients were, however, closely monitored in the hospital and may thus have reflected the minute change of vital signs.

As regards (2), “bilateral pneumonia” was described as an inclusion criterion in the clinical trial of tocilizumab, a monoclonal antibody against the interleukin-6 receptor [8]. In hamsters, an experimental infection with SARS-CoV-2 P.1 variant demonstrated ground glass opacity in microcomputed tomography as well as histopathological evidence of antigen in the bronchi and the alveoli on Day 7 [9]. Thus, tomographic findings of ground glass attenuation reflect the viral load of the alveolar cell, enhancing damage to the gas exchange mechanism.

These events, temperature increase and oximetry decrease, may reflect antibody-dependent enhancement (ADE) especially in vaccinees of SARS-C0V-2. In ADE, virus-specific antibodies can augment virulence [10]; experimentally, casirivimab and imdevimab together and imdevimab alone, but not casirivimab alone, mediated entry of virus-like particles pseudotyped with SARS-CoV-2 spike protein into immune cell lines [4]. Furthermore, enhanced respiratory disease (ERD) may lead to hypoxia by the use of convalescent sera or monoclonal antibodies [10]. Our study, however, showed that 2-time vaccination prevented post-infusion temperature elevation (Table 2). Albeit without statistical significance, 2-time vaccination showed tendency of preventing oximetry decrease as well (Table 3). Thus, it was unlikely that ADE and/or ERD caused fever and oximetry decrease.

As for bradycardia, few has been reported related to casirivimab-imdevimab except the Japanese authorization agency [1] referring to the possibility of AE as well as of worsening of COVID-19. Li and colleagues [11] reported that one participant receiving SCTA01, a monoclonal antibody targeting SARS-CoV-2, developed sinus bradycardia but recovered in 7 h. In an anti-viral drug, Hsu and others [12] described that remdesivir can cause atrioventricular block, leading into bradyarrhthmia. COVID-19 per se, however, has been reported to manifest arrhythmia including bradycardia [13]. Thus, causes of bradycardia in our series remain unsolved as to AE of the antibody cocktail or a symptom of COVID-19.

Limitations for this study included the difficulty distinguishing AE from worsening of COVID-19, thus we documented as clinical events. Further study is awaited to clarify the mechanism causing temporary fever and oximetry decrease.

## Conclusions

For 24 h after infusion of casirivimab-imdevimab, COVID-19 patients with increased white blood cell counts may be predisposed to temperature elevation more than 1.0 degrees centigrade, as may bilateral ground glass attenuation to lowered oximetry. While these events remain unclear as to post-infusion AE or COVID-19 worsening, patients with leukocytosis and bilateral ground glass attenuation may need precaution.

## Data Availability

The datasets used and/or analyzed during the current study are available from the corresponding author on reasonable request.

## Abbreviations

AE: adverse effects
BMI: body mass index
CT: computed tomography
ROC: receiver-operating characteristics
OR: odds ratio
CI: 95% confidence interval
ADE: antibody-dependent enhancement
ERD: enhanced respiratory disease

## References

[1] Weinreich DM, Sivapalasingam S, Norton T, Ali S, Gao H, Bhore R, Musser BJ, Soo Y, Rofail D, Im J, Perry C, Pan C, Hosain R, Mahmood A, Davis JD, Turner KC, Hooper AT, Hamilton JD, Baum A, Kyratsous CA, Kim Y, Cook A, Kampman W, Kohli A, Sachdeva Y, Graber X, Kowal B, DiCioccio T, Stahl N, Lipsich L, Braunstein N, Herman G, Yancopoulos GD, Trial Investigators (2021). REGN-COV2, a neutralizing antibody cocktail, in outpatients with Covid-19. N Engl J Med.

[2] Infectious Disease Society of America. Guideline. Immunomodulators. Last reviewed: September 3, 2021. https://www.idsociety.org/covid-19-real-time-learning-network/therapeutics-and-interventions/immunomodulators/. Accessed 18 Sep 2021.

[3] Pharmaceuticals and Medical Devices Agency. FACT SHEET FOR HEALTH CARE PROVIDERS EMERGENCY USE AUTHORIZATION (EUA) OF REGEN-COVTM (casirivimab and imdevimab) AUTHORIZED USE. https://www.pmda.go.jp/drugs/2021/P20210719001/450045000_30300AMX00310_B100_1.pdf. Accessed 18 Sep 2021.

[4] U.S. Food and Drug Administration. FACT SHEET FOR HEALTH CARE PROVIDERS EMERGENCY USE AUTHORIZATION (EUA) OF REGEN-COV® (casirivimab and imdevimab). https://www.fda.gov/media/145611/download. Accessed 18 Sep 2021.

[5] National Institute of Infectious Diseases of Japan. Current Situation of Infection, September 1, 2021. 50th meeting of the COVID-19 advisory board of Ministry of Health, Labour and Welfare (September 1, 2021). https://wwwniidgojp/niid/en/2019-ncov-e/10636-covid19-ab50th-enhtml. Accessed 18 Sep 2021.

[6] U.S. DEPARTMENT OF HEALTH AND HUMAN SERVICES. Common Terminology Criteria for Adverse Events (CTCAE) Version 5.0 Published: November 27, 2017. https://ctep.cancer.gov/protocoldevelopment/electronic_applications/docs/ctcae_v5_quick_reference_5x7.pdf. Accessed 18 Sep 2021.

[7] Liu XY, Lv FJ, Lv SX, Fu BJ, Md WL, Ouyang Y, Chu ZG (2021). Completed absorption of coronavirus disease 2019 (COVID-19) pneumonia lesions: a preliminary study. Int J Med Sci.

[8] Shang L, Lye DC, Cao B (2021). Contemporary narrative review of treatment options for COVID-19. Respirology..

[9] Imai M, Halfmann PJ, Yamayoshi S, Iwatsuki-Horimoto K, Chiba S, Watanabe T, Nakajima N, Ito M, Kuroda M, Kiso M, Maemura T, Takahashi K, Loeber S, Hatta M, Koga M, Nagai H, Yamamoto S, Saito M, Adachi E, Akasaka O, Nakamura M, Nakachi I, Ogura T, Baba R, Fujita K, Ochi J, Mitamura K, Kato H, Nakajima H, Yagi K, Hattori SI, Maeda K, Suzuki T, Miyazato Y, Valdez R, Gherasim C, Furusawa Y, Okuda M, Ujie M, Lopes TJS, Yasuhara A, Ueki H, Sakai-Tagawa Y, Eisfeld AJ, Baczenas JJ, Baker DA, O’Connor SL, O’Connor DH, Fukushi S, Fujimoto T, Kuroda Y, Gordon A, Maeda K, Ohmagari N, Sugaya N, Yotsuyanagi H, Mitsuya H, Suzuki T, Kawaoka Y (2021). Characterization of a new SARS-CoV-2 variant that emerged in Brazil. Proc Natl Acad Sci U S A.

[10] Munoz FM, Cramer JP, Dekker CL, Dudley MZ, Graham BS, Gurwith M, Law B, Perlman S, Polack FP, Spergel JM, van Braeckel E, Ward BJ, Didierlaurent AM, Lambert PH, Brighton Collaboration Vaccine-associated Enhanced Disease Working Group (2021). Vaccine-associated enhanced disease: case definition and guidelines for data collection, analysis, and presentation of immunization safety data. Vaccine..

[11] Li Y, Qi L, Bai H, Sun C, Xu S, Wang Y, Han C, Li Y, Liu L, Cheng X, Liu J, Lei C, Tong Y, Sun M, Yan L, Chen W, Liu X, Liu Q, Xie L, Wang X (2021). Safety, tolerability, pharmacokinetics, and immunogenicity of a monoclonal antibody (SCTA01) targeting SARS-CoV-2 in healthy adults: a randomized, double-blind, placebo-controlled. Phase I Study Antimicrob Agents Chemother.

[12] Hsu JY, Mao YC, Liu PY, Lai KL. Pharmacology and Adverse Events of Emergency-Use Authorized Medication in Moderate to Severe COVID-19. Pharmaceuticals (Basel). 2021;14:955. 10.3390/ph14100955 PMC8536968 34681179

[13] Umbrajkar S, Stankowski RV, Rezkalla S, Kloner RA (2021). Cardiovascular Health and disease in the context of COVID-19. Cardiol Res.

[14] Ethics Committee of Medical Research, Shimonoseki City Hospital: Adverse effect predictors for casirivimab/imdevimab. https://shimonosekicity-hosp.jp/content/100862153.pdf. Accessed 19 Oct 2021.

[15] Ministry of Education, Culture, Sports, Science and Technology and the Ministry of Health, Labour and Welfare, Japan. Ethical Guidelines for Medical and Health Research Involving Human Subjects, Provisional Translation (as of July 2018) https://www.lifescience.mext.go.jp/files/pdf/n2181_01.pdf. Accessed 18 Sep 2021.

